# Intratumoral heterogeneity of second-harmonic generation scattering from tumor collagen and its effects on metastatic risk prediction

**DOI:** 10.1186/s12885-020-07713-4

**Published:** 2020-12-10

**Authors:** Danielle E. Desa, Robert L. Strawderman, Wencheng Wu, Robert L. Hill, Marcel Smid, J. W. M. Martens, Bradley M. Turner, Edward B. Brown

**Affiliations:** 1 Department of Biomedical Engineering, Hajim School of Engineering and Applied Sciences, University of Rochester, Rochester, New York USA; 2 Department of Biostatistics and Computational Biology, School of Medicine and Dentistry, University of Rochester Medical Center, Rochester, New York USA; 3 Goergen Institute for Data Science, University of Rochester, Rochester, New York USA; 4 Harmonigenic Corporation, Rochester, New York USA; 5 Department of Medical Oncology, Erasmus MC Cancer Institute, Erasmus University Medical Center, Rotterdam, Netherlands; 6 Department of Pathology and Laboratory Medicine, School of Medicine and Dentistry, University of Rochester Medical Center, Rochester, New York USA

**Keywords:** Breast cancer, Metastasis, Second-harmonic generation, Multiphoton microscopy, Collagen, Tumor microenvironment, F/B, Prognosis

## Abstract

**Background:**

Metastases are the leading cause of breast cancer-related deaths. The tumor microenvironment impacts cancer progression and metastatic ability. Fibrillar collagen, a major extracellular matrix component, can be studied using the light scattering phenomenon known as second-harmonic generation (SHG). The ratio of forward- to backward-scattered SHG photons (F/B) is sensitive to collagen fiber internal structure and has been shown to be an independent prognostic indicator of metastasis-free survival time (MFS). Here we assess the effects of heterogeneity in the tumor matrix on the possible use of F/B as a prognostic tool.

**Methods:**

SHG imaging was performed on sectioned primary tumor excisions from 95 untreated, estrogen receptor-positive, lymph node negative invasive ductal carcinoma patients. We identified two distinct regions whose collagen displayed different average F/B values, indicative of spatial heterogeneity: the cellular tumor bulk and surrounding tumor-stroma interface. To evaluate the impact of heterogeneity on F/B’s prognostic ability, we performed SHG imaging in the tumor bulk and tumor-stroma interface, calculated a 21-gene recurrence score (surrogate for OncotypeDX®, or S-ODX) for each patient and evaluated their combined prognostic ability.

**Results:**

We found that F/B measured in tumor-stroma interface, but not tumor bulk, is prognostic of MFS using three methods to select pixels for analysis: an intensity threshold selected by a blinded observer, a histogram-based thresholding method, and an adaptive thresholding method. Using both regression trees and Random Survival Forests for MFS outcome, we obtained data-driven prediction rules that show F/B from tumor-stroma interface, but not tumor bulk, and S-ODX both contribute to predicting MFS in this patient cohort. We also separated patients into low-intermediate (S-ODX < 26) and high risk (S-ODX ≥26) groups. In the low-intermediate risk group, comprised of patients not typically recommended for adjuvant chemotherapy, we find that F/B from the tumor-stroma interface is prognostic of MFS and can identify a patient cohort with poor outcomes.

**Conclusions:**

These data demonstrate that intratumoral heterogeneity in F/B values can play an important role in its possible use as a prognostic marker, and that F/B from tumor-stroma interface of primary tumor excisions may provide useful information to stratify patients by metastatic risk.

**Supplementary Information:**

The online version contains supplementary material available at 10.1186/s12885-020-07713-4.

## Background

Breast cancer is the most common invasive cancer in women, with the majority of deaths attributed to metastasis [1]. Breast tumors are typically classified using molecular and genetic markers as well as clinical staging systems. These biomarkers are analyzed to determine prognosis, predict response to therapies, and are also used as surrogates for outcome (i.e. measures of treatment effects that correlate with a clinical endpoint) in invasive breast cancers [2]. Such characterization may include expression levels of estrogen receptor (ER), progesterone receptor, and human epidermal growth factor receptor-2, and testing for other proliferation, invasion, and epithelial-mesenchymal transition markers. Tumors can exhibit heterogeneity, with different regions expressing different genetic aberrations [3–6] and differences in key biomarkers [7]. Heterogeneity in cell types is frequently seen in breast cancers and has been linked to poor patient prognosis independent of typical clinical variables including ER status, lymph node involvement, and tumor size [8]. Improving individualized treatments and overall patient survival therefore requires a better understanding of heterogeneity in the breast tumor microenvironment [9, 10]. Likewise, developing any new biomarker requires an understanding of its heterogeneity within tumors and the impact of that heterogeneity on its clinical utility.

Both cellular and noncellular components of the microenvironment facilitate primary tumor growth and metastasis [3, 11–13]. The ECM is an essential component of the solid tumor microenvironment, affecting cell biomechanics and signaling and therefore directly impacting metastatic potential. Microscopic ECM properties provide biophysical support and chemical cues necessary for normal cell function and are a result of constant matrix synthesis, modification, and degradation [14, 15]. As a major component of the ECM, collagen plays a critical role in cell migration and differentiation [14, 16]. Increased collagen deposition and crosslinking are associated with malignancy, and changes in collagen organization are thought to promote tumor cell invasion, possibly via in increased protein deposition, increased tissue stiffness, and linearization of collagen fibers [17–24]. In more aggressive breast cancers, irregular tumor-stroma boundaries are seen with orthogonally-aligned fibers facilitating cell invasion [18]. These altered collagen properties in turn affect biochemical signaling and tissue biomechanical properties, encouraging tumor cell proliferation, migration, and dysregulation of normal cellular activities [25].

Fibrillar collagen can produce an intrinsic optical signal called second-harmonic generation (SHG) [26]. SHG is a nonlinear optical phenomenon that occurs when two identical photons scatter off a noncentrosymmetric material, producing a single photon with exactly twice the energy of the initial photons [26]. In SHG images of excised primary breast tumor, the presence of collagen fibers oriented perpendicular to the tumor border has been shown to be prognostic of breast cancer progression and to enable tumor cell invasion [19, 27, 28], and in in vivo models of breast cancer, tumor cells can be observed locomoting along SHG+ collagen fibers [29]. Recent studies have demonstrated that SHG can be used, alone and in conjunction with two-photon excited fluorescence, to identify early stages of breast ductal carcinoma, collagen morphological changes during tumor progression, and to provide prognostic information on patient survival [30–32].

In addition to producing images whose properties provide insight into disease states, SHG polarization and scattering directionality can reveal important information about tissue. Polarization-resolved SHG has also been used to assess structural changes in ECM collagen during disease progression, including breast cancers [33–36]. This technique exploits the polarization of incident light to reveal information about collagen at the molecular level including helix pitch angles, fibril organization, and orientation. The directionality of SHG scattering from an individual fiber is sensitive to its internal structure, including fibril diameter, spacing, and disorder of fibril packing within the fiber [37–40]. We collectively call these three parameters the collagen fiber internal structure (FIS). One measure of SHG emission directionality is the ratio of forward-emitted to backward-emitted SHG (where “forward” is in the direction of the excitation laser), or the F/B ratio. F/B is sensitive to FIS, can be measured on a point-by-point basis or used to generate F/B images, and is distinct from the overall orientation of a fiber in an SHG image [19, 41]. SHG directionality imaging and analysis has been used to distinguish healthy and diseased tissue in breast [42, 43], ovarian [44], lung [45], and basal cell cancers [46].

We have previously shown that the average F/B from SHG images of a cohort of untreated ER+, lymph node negative (LNN), invasive ductal carcinoma (IDC) samples is an independent prognostic indicator of metastasis-free survival time (MFS) [47]. The samples used in that study were tissue microarrays comprised of 1-mm tissue discs and as such, the F/B values prognostic of MFS in that study were taken from a small part of the excised primary tumor.

Recently, we performed SHG imaging on core needle biopsy sections taken from breast cancer patients prior to neoadjuvant chemotherapy (NACT) administration. As these tissue strips (~ 0.2 × 1.5 cm) span multiple regions of interest (ROIs), we selected two types of regions for study: the cellular tumor bulk and the surrounding tumor-stroma interface, consisting mainly of ECM proteins and stromal cells, each identified by a clinical pathologist. We found that F/B measured in the tumor-stroma interface, but not tumor bulk, was associated with Residual Cancer Burden class, one measure of NACT response [48]. These results from needle biopsy sections revealed that heterogeneity in collagen FIS affects the relationship between pre-treatment F/B and subsequent NACT response.

Prompted by that discovery, in this study we investigated how heterogeneity in primary tumor excisions affects the ability of F/B to predict MFS in untreated IDC patients. We evaluated the association between collagen FIS (as reported by F/B) and MFS in two regions associated with tumor tissue in primary tumor excisions. We then used both regression trees [49] and Random Survival Forests (RSF) [50] to further explore this association and the combined prognostic ability of F/B and a widely used 21-gene recurrence score. Our results reveal that heterogeneity in SHG F/B measurements within breast tumor samples is significant and must be considered when evaluating this method as a possible predictor of metastasis and further suggest that F/B measured in appropriate tumor regions may add prognostic information to currently used genomic methods.

## Methods

### Patient samples

Slides prepared from post-biopsy primary breast cancer surgical excisions from 95 different patients were used from a collection at the Erasmus Medical Center (Rotterdam, Netherlands). The studies on secondary use of archived tissues was approved in writing by the Medical Ethics Committee of the Erasmus Medical Center, Rotterdam, Netherlands (MEC 02.953) and was performed in accordance with the Code of Conduct (The Code for Proper Secondary Use of Human Tissue) of the Federation of Medical Scientific Societies in The Netherlands. Primary tumor excisions were formalin-fixed and paraffin-embedded, (FFPE) mounted on slides as 5 μm sections and stained with hematoxylin and eosin (H&E). Multiple regions were imaged within a single 5 μm section from each patient in this study. Patients were tested for ER status using immunohistochemistry, where the cutoff for receptor positivity was 10% positive tumor cells. All patients were ER+ and LNN and had not been treated with NACT or adjuvant hormonal nor chemotherapy. Other primary tumor characteristics are summarized in Supplementary Table 1. Some patients received radiation therapy, which has been shown not to affect distant metastases [51]. No other treatment was received before nor after excision of the primary tumor. We note that these are historical samples and clinical practice in the Netherlands at the time was more focused on monitoring patients. Thus, these cases are uniquely suitable for a purely prognostic study.

Follow-up data on patient outcomes were recorded every 3 months for 2 years, every 6 months for years 3–5, and every 12 months afterwards. Gene expression data for these patients are archived in the GEO repository (ncbi.nlm.nih.gov/geo/) as part of databases GSM2034 and GSM5327. In order to study F/B in the context of the 21-gene OncotypeDX® score, we used a surrogate 21-gene score (S-ODX) calculated from these gene expression data using the publicly available Recurrence Online tool (www.recurrenceonline.com), an online analysis tool to determine breast cancer recurrence scores and hormone receptor status using microarray data [52].

In this study, we wanted to evaluate the effect of heterogeneity on F/B’s prognostic ability in a clinical setting, with the hope of producing a clinically relevant technique for predicting metastasis. Therefore, we performed SHG imaging and F/B analysis using samples that were already within the clinical workflow, specifically the typical FFPE H&E sections generated from primary tumor excisions, as opposed to fresh, unprocessed tumor tissue. Using these FFPE H&E sections also allows us to easily identify clinically relevant regions within the tumor that inform pathologists’ diagnoses and recommendations. Consequently, we note that the F/B value we report here is not necessarily equal to F/B that would be measured in unprocessed fresh tissue because various steps in processing and mounting may affect the F/B value.

### Imaging

A Spectra Physics MaiTai Ti:Sapphire laser (circularly polarized at the sample using a Berek Compensator, 100 fs pulses at 80 MHz, 810 nm, ~ 4 mW at the sample) was directed through an Olympus Fluoview FV300 scanner. The laser was focused through an Olympus UMPLFL20XW water-immersion lens (20x, 0.95 NA), which subsequently captured backward-propagating SHG signal (i.e. the B image). This backward-propagating SHG signal was separated from the excitation beam using a 670 nm dichroic mirror, filtered (HQ405/30 m-2P, Chroma), and collected by a photomultiplier tube (Hamamatsu H10492–003). The forward-scattered SHG (i.e. the F image) was collected through an Olympus 0.9 NA condenser, reflected by a 565 nm dichroic mirror (565 DCSX, Chroma) to remove excitation light, and captured using an identical filter (HQ405/30 m-2P, Chroma) and identical photomultiplier tube (Hamamatsu H10492–003) with minimal autofluorescence captured, as previously described [48].

### Image analysis

#### User-defined thresholds

Image pairs were analyzed using Fiji, as we have previously described [47, 48, 53]: in summary, to produce a forward-to-backward scattering ratio (F/B) for a given ROI, two masks (one for the F image and one for the B image) were created by a blinded observer selecting a threshold for each F and each B image that best distinguished pixels within fibers from background pixels. Pixels above the threshold were set to 1 and those below to 0, producing binary F and B masks. The binary masks were multiplied together to create a final mask of pixels within collagen fibers. In this final mask, pixels are assigned a value of 1 only if the value of that pixel is 1 in both the F and B masks (i.e. located within a collagen fiber), and 0 if otherwise. The background-subtracted F and B images were divided to produce a single F/B image, which was multiplied by this final mask (the product of the binary F and B masks). The average value of the nonzero pixels from the resultant image yielded the average F/B of the entire ROI.

#### Histogram-based thresholding

To reduce possible user bias in image analysis, histogram-based automatic image thresholding (“Otsu’s method”) was also performed [54]. This method separates pixels into two classes: foreground pixels above a selected threshold (i.e. collagen fiber) and background pixels. The algorithm steps through all possible thresholds, calculates the variance of each pixel class and selects the threshold that minimizes the sum of foreground and background variances. A threshold was selected for each image using Otsu’s method with a scaling factor of 0.6 implemented in MATLAB (Mathworks, Inc.). Foreground pixels were converted to 1 and background to 0 to produce binary F and B masks, which were multiplied by the background-subtracted F/B image to produce a single F/B value as described above.

#### Adaptive thresholding

While histogram-based segmentation succeeds in calculating thresholds with minimal user input (reducing possible bias), this method does not consider heterogeneity (i.e. spatial variation in intensity) within the images. A real-time adaptive thresholding method was used to account for these variations as follows [55]. Our goal continues to be to generate a mask whose pixel values are 1 if they are in collagen fibers and 0 if they are background pixels. In adaptive thresholding, we do this by considering each pixel in the image and assigning that pixel a value of 1 or 0, depending on its value relative to the average intensity of the pixels in a window centered on the pixel in question. A small window size is desirable as it will be more responsive to variations in image intensity, allowing somewhat dim collagen pixels in regions whose collagen is overall very dark to be assigned a value of 1. However, if the window is too small, it could be contained within regions that are entirely collagen-free, thus assigning background pixels a value of 1. Hence our first task is to identify the smallest window size for each image that will *not* fit entirely into collagen-free regions. For this initial task we first binarize the entire image based upon whether each pixel is greater than or less than an arbitrary value (0.6x the average pixel intensity of the entire image). Then, a series of progressively smaller windows are scanned across the binarized image and the percentage of nonzero pixels calculated for every possible position of the window in the image. The first window size that, when applied to any location in the image, has < 5% nonzero pixels is selected as the smallest allowed window size for that image. This window size is next applied to the original image and a mask is generated whereby each pixel in the image is assigned a value of 1 or 0 depending upon whether it is less than or greater than 0.6x the average pixel value in the window surrounding it. This algorithm was implemented in MATLAB (Mathworks, Inc.) and a binary mask produced for each image. The resultant F and B masks were then multiplied by the background-subtracted F/B image to produce a single F/B value as described above.

All graphs were generated using GraphPad Prism 5 and statistical analysis performed using Prism 5 or R.

#### Calibration

For each imaging session, an F and B image of no sample (for background) and a reference F and B image of a dilute stock solution of fluorescein isothiocyanate (FITC) were collected (to quantify day-to-day variations in laser alignment, detector alignment, etc.). Variations were normalized by dividing each patient sample’s F/B value by F/B of the measured FITC value for that imaging session.

## Results

**Fig. 1:**
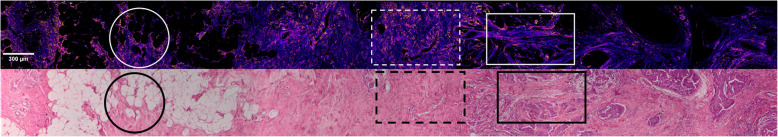
Collagen F/B is heterogeneous within primary breast tumor tissue. SHG F/B images (a series of adjacent ROIs extending along the *x*-axis) and matching H&E images were stitched end-to-end to form a composite ROI for a representative sample. Examples of tumor bulk (solid box), tumor-stroma interface (dashed box), and uninvolved tissue (circle) are shown. The F/B values were plotted in a false color “heatmap” (low F/B values = dark blue, high F/B values = yellow) to illustrate differences in F/B within the tumor region including tumor bulk and tumor-stroma interface, indicating intratumoral heterogeneity in F/B

To illustrate the heterogeneity in collagen FIS, we sequentially acquired H&E and SHG images from one sample by holding the *y* position of the ROI constant and moving across the tissue section in *x*. The resultant series of images (typically 660 × 660 μm ROIs for SHG) were stitched together to form image strips (Fig. 1 and Supplementary Fig. 1) [56]. In the H&E image, our collaborating breast pathologist marked different regions including the tumor bulk and surrounding tumor-stroma interface (solid and dashed boxes, respectively) as well as uninvolved tissue (circle). To produce the accompanying F/B image we calculated F/B in the SHG strip using user-defined thresholds to set background pixels to zero as described above. Variations in collagen FIS, as reported by F/B, are clearly visible in both the tumor bulk (solid box) and tumor-stroma interface (dashed box).

### Collagen fiber internal structure differs between tumor regions

**Fig. 2:**
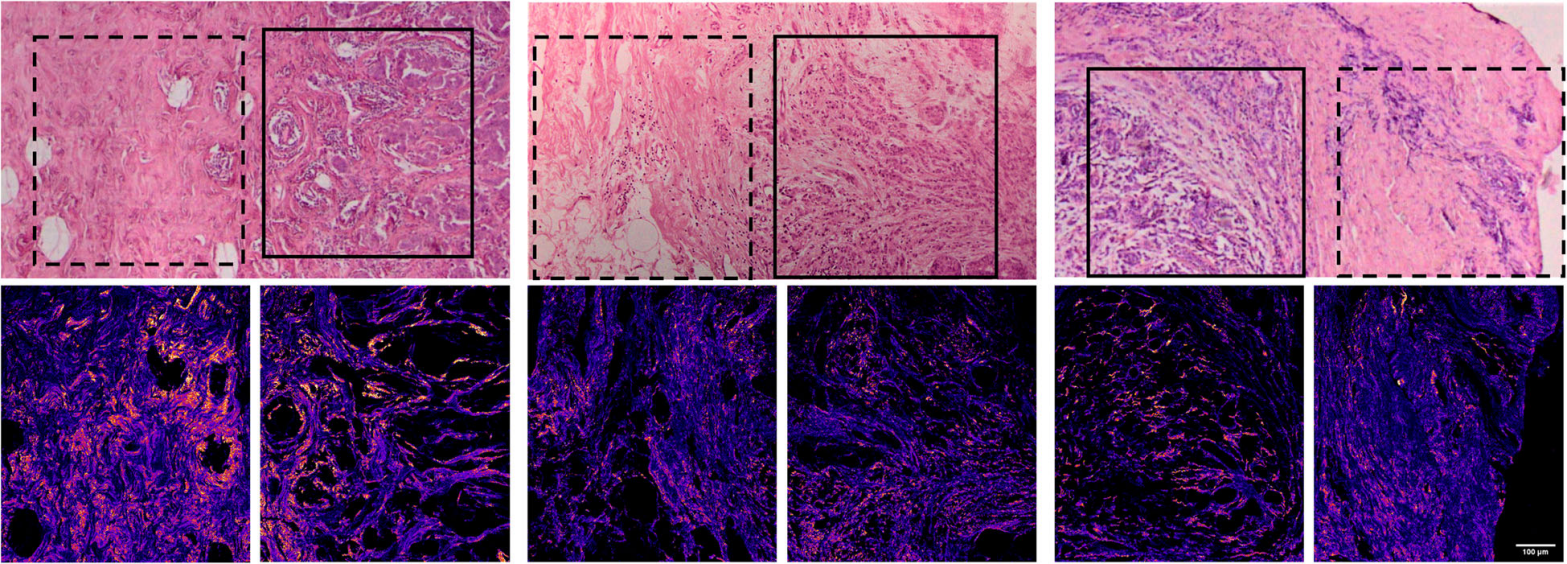
Collagen features vary between the tumor bulk and the tumor-stroma interface of primary tumor excisions. Primary tumor excisions contain both tumor bulk (solid boxes) and tumor-stroma interface (dashed boxes). Tumor bulk consists of tumor cell clusters surrounded by individual SHG-producing fiber bundles. The tumor-stroma interface is comprised mainly of closely packed collagen fibers and individual stromal cells adjacent to the tumor bulk. Representative SHG F/B and matching H&E images from 3 individual patients are shown. The scale bar applies to all images in this figure

We next investigated whether the observed variations in F/B can be found systemically between biologically relevant, identifiable regions in the tumor. In collaboration with breast pathologists, we previously identified two region types in breast cancer core needle biopsies: the highly cellular tumor bulk and the tumor-stroma interface directly adjacent (i.e. within ~ 660 μm, one microscope field of view), which is comprised mainly of ECM proteins. In that study of core needle biopsies, we observed that F/B from the tumor-stroma interface, but not the tumor bulk, of core needle biopsy sections is associated with NACT response in certain breast cancer patients [48]. As a result, in this study we chose to evaluate F/B in the same two types of regions (tumor bulk and tumor-stroma interface) in sections from primary tumor excisions. Examples of these regions are shown in both H&E and SHG in Fig. 2.

**Fig. 3:**
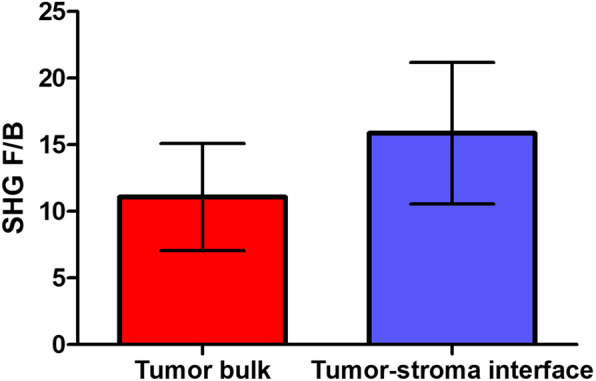
F/B in tumor-stroma interface versus tumor bulk. Collagen fiber internal structure, as represented by F/B, is significantly different between tumor bulk and the tumor-stroma interface in IDC ER+ LNN excised primary tumors. Error bars = SD, *t*-test, *p* < 0.0001, *n* = 92

An observer blinded to patient outcome was trained by a pathologist to recognize these regions in primary excisions and acquired 3 images in the tumor bulk and 3 in the tumor-stroma interface of each slide. The F/B values for each of the 3 ROIs within each region type were averaged to produce a single F/B for tumor bulk and one for tumor-stroma interface in each patient. In most of the excisions (*n* = 92 of 95), a clear tumor-stroma interface was available for imaging. In agreement with our previous results for core needle biopsy samples imaged prior to NACT [48], we found a significant difference between measured bulk and interface F/B values, indicating heterogeneity in collagen FIS between these two regions of the tumor (*p* < 0.0001, Fig. 3). Additionally, to put these values in context we imaged further away from tumor bulk (at least one full field of view, ~ 660 μm). This was possible in 60 of the 95 slides, and an example region is circled in Fig. 1. The F/B values from these “far” regions (17.7 ± 6.60) were significantly greater than the tumor bulk (t-test, *p* < 0.0001, data not shown) and tended to be slightly greater than the tumor-stroma interface (t-test, *p* = 0.07, data not shown).

### F/B generated from user-defined thresholds and its relation to metastasis-free survival

Our previous discovery that F/B is an independent prognostic indicator of metastasis-free survival time was generated using a tissue microarray in which 1-mm diameter discs were available for each patient and minimal information on intratumoral heterogeneity was obtainable [47]. Armed with larger tissue sections and the realization that F/B significantly varies between different tumor regions, one naturally then asks if assessing F/B in certain regions can improve its prognostic ability. Based on the observed significant FIS differences between the tumor bulk and tumor-stroma interface, we assessed the prognostic ability of F/B measured in each of those tumor regions.

**Fig. 4:**
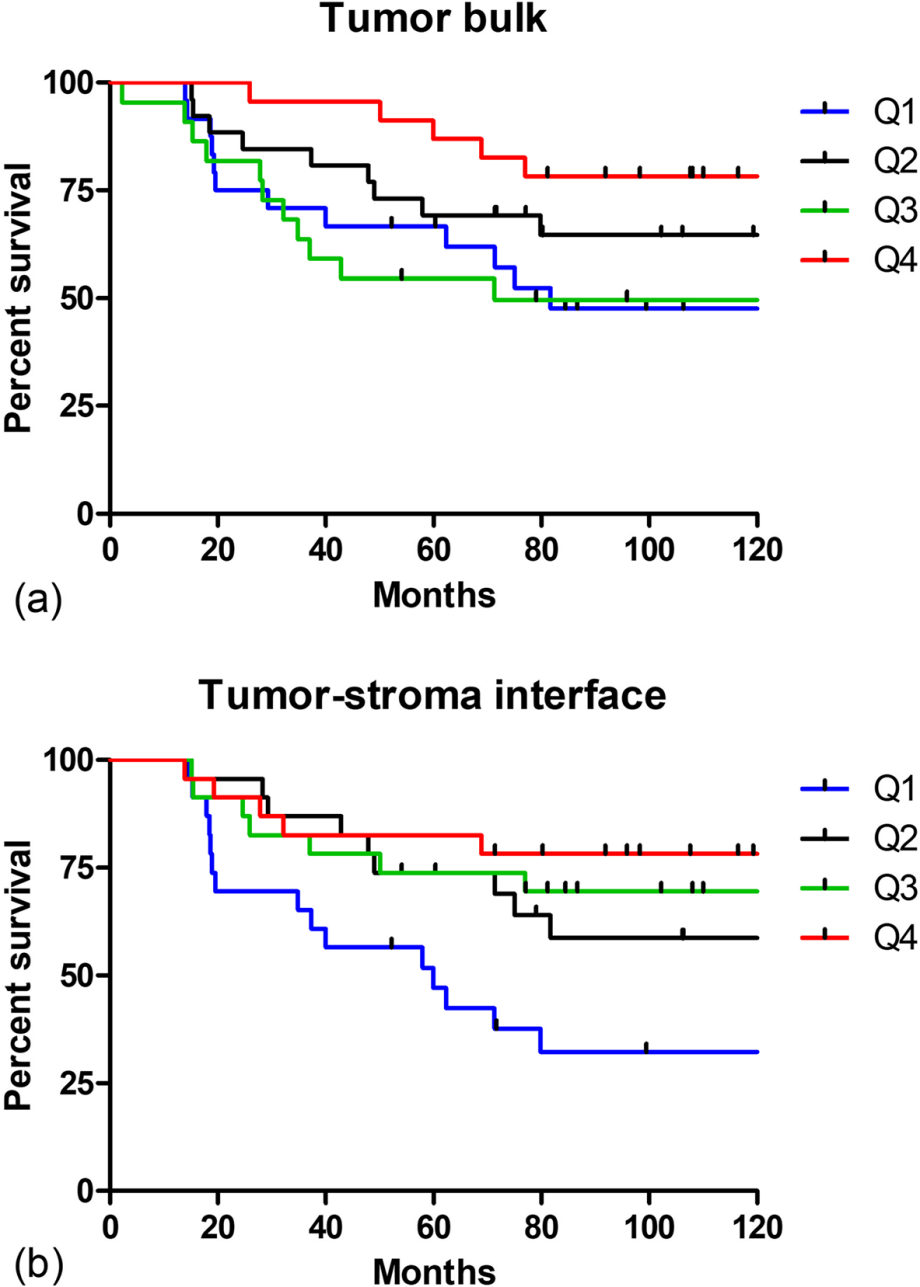
F/B measured in tumor-stroma interface and bulk of primary tumor sections and relation to MFS. SHG F/B values were produced by a user-defined threshold for each individual image from the **a**, tumor bulk and **b**, tumor-stroma interface. Patients were split into four equal quartiles (Q1 = lowest F/B) based on F/B, and the percentage of each quartile surviving without metastasis then plotted versus time. Tick marks represent censoring events caused when a patient dies of a cause other than cancer or is lost to follow-up. Partial likelihood ratio tests for ln F/B: *p* = 0.05 (tumor bulk, *n* = 95) and *p* = 0.00008 (tumor-stroma interface, n = 92)

Patients were first listed in order from lowest to highest based on ln F/B (natural log of F/B) measured in tumor bulk and then divided into four equal groups based on this ordering. The first group (Quartile 1, or Q1) contained patients with the lowest F/B (corresponding to values < 2.11, *n* = 24). Q2 consisted of patients with ln F/B from 2.11–2.35 (n = 24), Q3 with ln F/B from 2.35–2.56 (n = 24) and Q4 with the highest ln F/B values, > 2.56 (*n* = 23). A Kaplan-Meier plot was then generated for the tumor bulk (Fig. 4). A second plot (Fig. 4) was generated for ln F/B derived from the tumor-stroma interface in a similar manner (Q1 ln F/B: < 2.43, Q2 ln F/B: 2.43–2.69, Q3 ln F/B: 2.69–2.97, Q4 ln F/B: > 2.97, n = 23 in each quartile). Tests for a linear association between ln F/B and the log-relative risk of MFS were first carried out by fitting separate Cox regression models relating MFS to each of these two measures, with corresponding partial likelihood ratio test *p*-values of 0.05 (tumor bulk) and 0.0008 (tumor-stroma interface). A Cox regression model including both measures was additionally fit to these same data and demonstrated no effect of ln F/B for tumor bulk (*p* = 0.63) when simultaneously accounting for ln F/B from the tumor-stroma interface (*p* = 0.0046). In all cases, the regression models demonstrate an empirical trend of decreasing relative risk with increasing F/B, whether taken from tumor bulk or tumor host-interface. Taken in combination, these results suggest that F/B from the tumor-stroma interface has a stronger explanatory effect for MFS than does F/B from the tumor bulk.

### F/B generated using histogram-based thresholding and its relation to metastasis-free survival

One possible reason for the different prognostic ability of F/B from tumor-stroma interface versus tumor bulk is that the two regions appear differently to the outcome-blinded observer who must choose intensity thresholds to select bright pixels within collagen fibers and reject dark pixels in background regions. For example, regions in the tumor bulk typically contain well-defined individual fiber bundles (solid boxes, Fig. 2) while regions in the tumor-stroma interface typically contain closely packed collagen fibers (dashed boxes, Fig. 2). The observer’s selection of thresholds may be influenced by these differing image features, possibly affecting the resultant F/B and hence its prognostic ability.

**Fig. 5:**
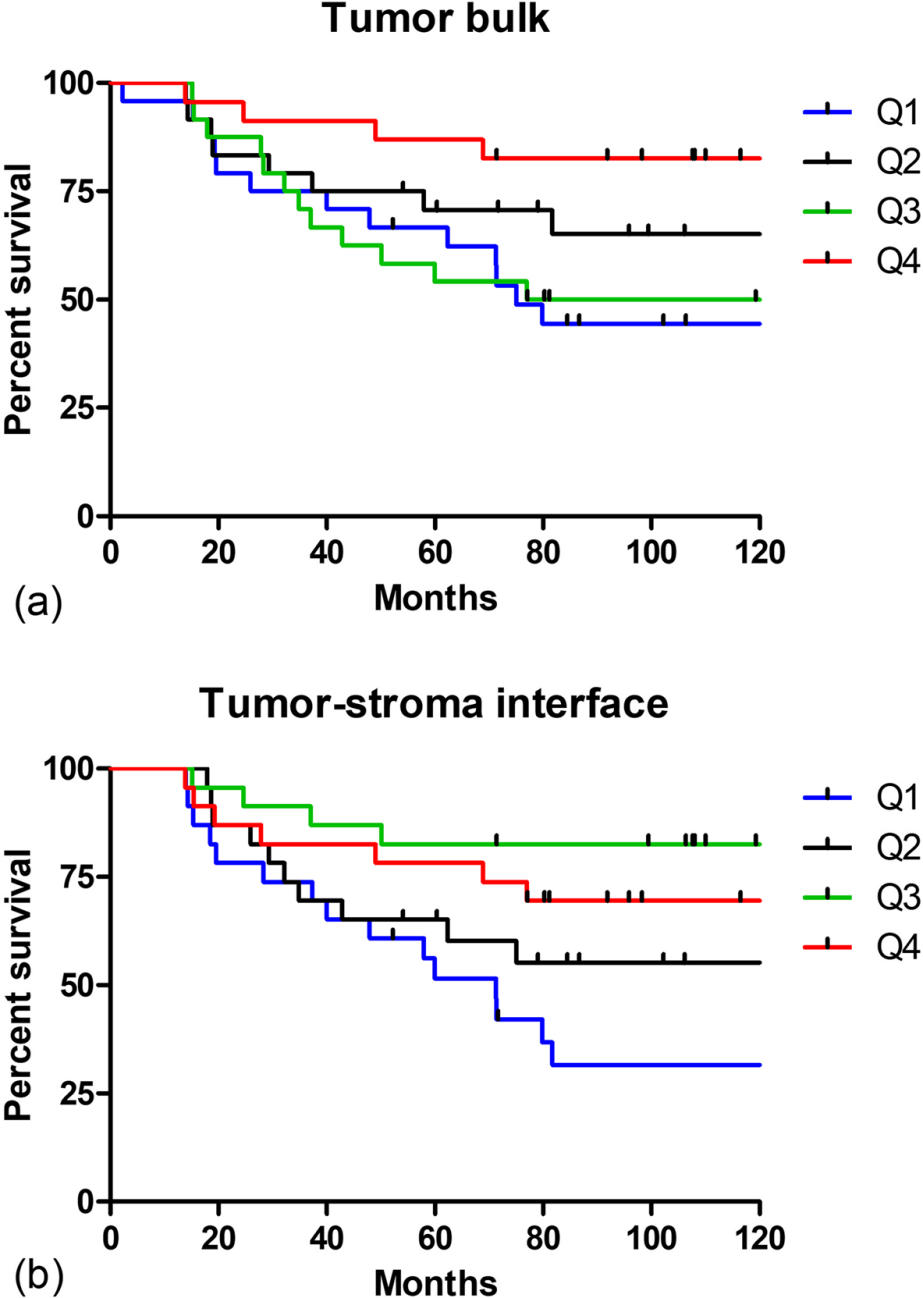
F/B generated using histogram-based thresholding and its relation to MFS. SHG F/B values were produced using binary masks generated by a histogram-based thresholding method for each individual image taken in the **a**, tumor bulk and **b**, tumor-stroma interface. Patients were split into four equal quartiles (Q1 = lowest F/B) based on F/B, and the percentage of each quartile surviving without metastasis then plotted versus time. Tick marks represent censoring events caused when a patient dies of a cause other than cancer or is lost to follow-up. Partial likelihood ratio tests for ln F/B: *p* = 0.01 (tumor bulk, *n* = 95) and *p* = 0.0009 (tumor-stroma interface, *n* = 92)

To reduce this possible user bias in calculating F/B, we next generated masks for distinguishing collagen pixels from background pixels using two less subjective techniques. First, a histogram-based technique (Otsu’s method) was used to separate pixels into foreground (collagen fibers) and background and generate a resulting binary mask. These masks were then applied to the background-subtracted F and B images and average F/B values were calculated as described above. We again found a significant difference between F/B calculated from tumor bulk versus tumor-stroma interface, indicating FIS heterogeneity between two regions of the tumor (*t*-test, *p* < 0.0001, *n* = 92). Patients were then divided into four quartiles based on ln F/B from each region (tumor bulk: Q1 ln FB: < 2.73, *n* = 24; Q2 ln F/B: 2.73–3.40, *n* = 24; Q3 ln F/B: 3.40–3.75, *n* = 24; Q4 ln F/B: > 3.75, *n* = 23; and tumor-stroma interface: Q1 ln F/B: < 3.37, Q2 ln F/B: 3.37–3.64, Q3 ln F/B: 3.64–3.97, Q4 ln F/B: > 3.97, *n* = 23 in each quartile) and Kaplan-Meier plots were generated (Fig. 5). As before, tests for a linear association between ln F/B and the log-relative risk of MFS were carried out by fitting separate Cox regression models respectively relating MFS to F/B from tumor bulk (*p* = 0.01) and tumor-stroma interface (*p* = 0.0009). The Cox regression model including both measures demonstrated no effect of ln F/B for tumor bulk (*p* = 0.50) when simultaneously accounting for ln F/B from the tumor-stroma interface (*p* = 0.0058). In all cases, the regression models again demonstrate an empirical trend of decreasing relative risk with increasing F/B, whether taken from tumor bulk or host-interface. When taken in combination, these results continue to suggest that F/B from the tumor-stroma interface has a stronger explanatory effect.

### F/B generated using adaptive thresholding and its relation to metastasis-free survival

**Fig. 6:**
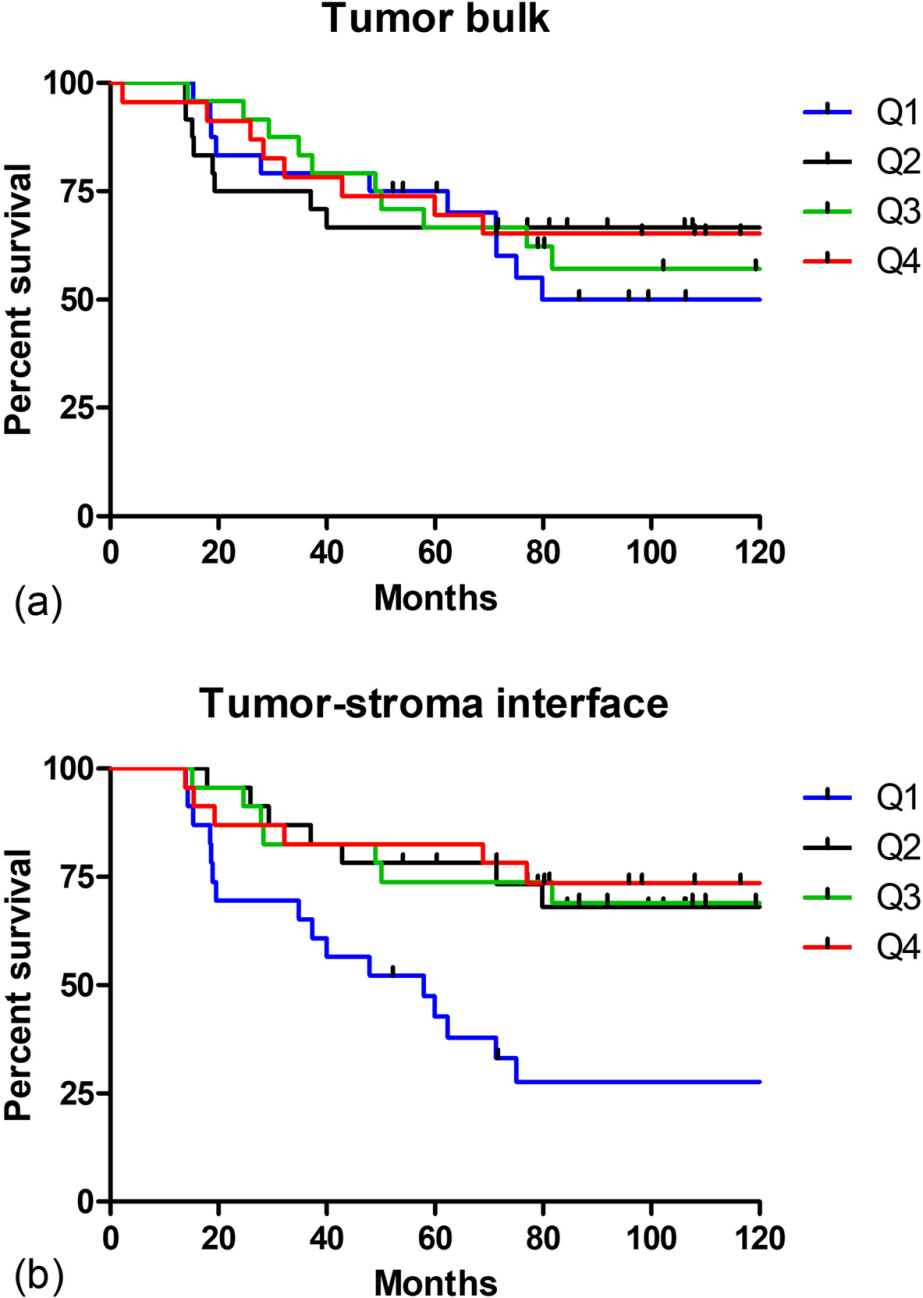
F/B generated using adaptive thresholding and its relation to MFS. An adaptive thresholding method was used to create binary masks used to calculate SHG F/B in the **a**, tumor bulk and **b**, tumor-stroma interface. Patients were split into four equal quartiles (Q1 = lowest F/B) based on F/B, and the percentage of each quartile surviving without metastasis then plotted versus time. Tick marks represent censoring events caused when a patient dies of a cause other than cancer or is lost to follow-up. Partial likelihood ratio test *p* = 0.4 (tumor bulk, *n* = 95) and *p* = 0.002 (tumor-stroma interface, *n* = 92)

While Otsu’s method succeeds in reducing the influence that an observer has on the process of selecting pixels for analysis, it does not account for heterogeneity in intensity within images, which may be critical when assessing heterogeneity in tumor ECM. Therefore, we next used an adaptive thresholding method that compares each pixel to the average of a surrounding window to determine its contribution to the binary mask. We calculated F/B using these masks and again found a significant difference between average F/B in tumor bulk and tumor-stroma interface (*t*-test, *p* < 0.0001, *n* = 92). Patients were divided into four quartiles based on ln F/B from each region (tumor bulk: Q1 ln F/B: < 2.01, *n* = 24; Q2 ln F/B: 2.01–2.39, *n* = 24; Q3 ln F/B: 2.39–2.75, *n* = 24; Q4 ln F/B: > 2.75, *n* = 23; and tumor-stroma interface: Q1 ln F/B: < 2.68, Q2 ln F/B: 2.68–2.93, Q3 ln F/B: 2.93–3.17, Q4 ln F/B: > 3.17, n = 23 in each quartile) and Kaplan-Meier plots were generated (Fig. 6). Tests for a linear association between ln F/B and the log-relative risk of MFS were again carried out by fitting separate Cox regression models respectively relating MFS to F/B from tumor bulk (*p* = 0.4) and tumor-stroma interface (*p* = 0.002). The Cox regression model including both measures demonstrated no effect of ln F/B for tumor bulk (*p* = 0.9) when simultaneously accounting for ln F/B from the tumor-stroma interface (*p* = 0.0028). The regression models demonstrate an empirical trend of decreasing relative risk with increasing F/B from the tumor host-interface. The combined results once again suggest that F/B from the tumor-stroma interface has a stronger explanatory effect.

### F/B and 21-gene recurrence scores

**Fig. 7:**
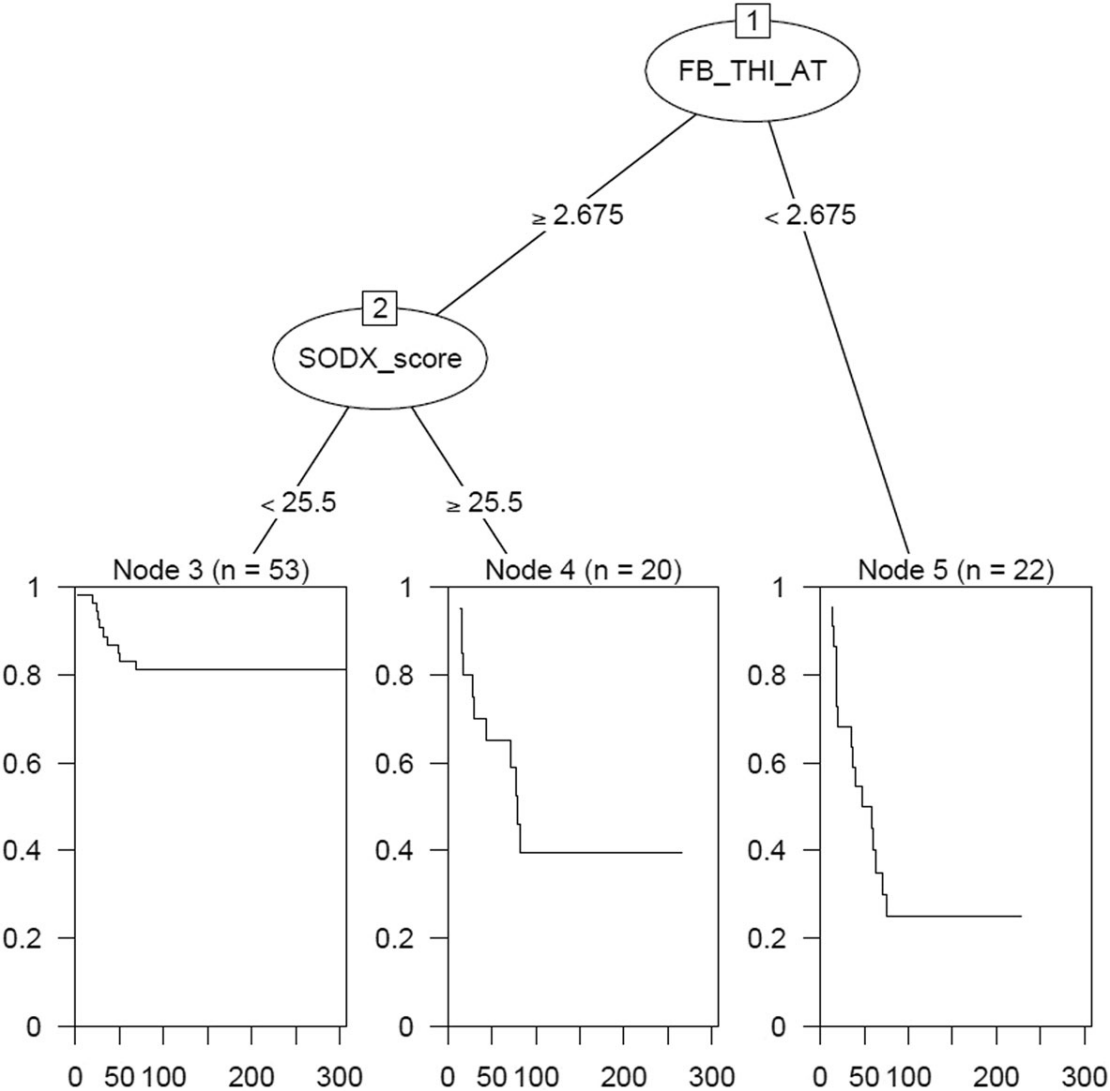
Regression tree derived using the method of Leblanc & Crowley. When given the SODX score and all six methods of generating F/B as inputs, this algorithm selects F/B from the tumor-stroma interface, calculated using the adaptive thresholding method (“FB_THI_AT”), and SODX score (“SODX_score”), as predictors of MFS. The RSF method (results not shown) identifies the same two predictors has having the highest variable importance in predicting MFS

One possible clinical use of F/B measurements would be to identify people who are at risk for experiencing future metastases and are therefore candidates for adjuvant therapy, should be considered for clinical trials, etc. The standard of care for the IDC ER+ LNN patient cohort at our institution (University of Rochester Medical Center) uses the 21-gene recurrence score assay OncotypeDX®. Using the latest post- Trial Assigning Individualized Options for Treatment (TAILORx) criterion, patients with a recurrence score of 26 and above are recommended for adjuvant chemotherapy while patients with a score of below 26 are not [57]. In order to study F/B in the context of OncotypeDX®, we used the Recurrence Online tool that uses gene expression data archived online in the GEO repository to calculate a surrogate 21-gene score (S-ODX) [52]. To evaluate the effect of heterogeneity on the ability of F/B to predict metastasis in conjunction with the S-ODX score, we utilized two related approaches to analyze these data: regression trees, and a Random Survival Forest (RSF) algorithm, which both derive a data-driven predictive regression model [50, 58–60]. In this case, both algorithms constructed a predictive model for MFS considering as input parameters the S-ODX score, F/B from tumor bulk calculated with the three image analysis methods, and F/B from tumor-stroma interface calculated with the three image analysis methods. The regression tree approach selected two of the input parameters (S-ODX and tumor-stroma interface F/B as calculated using adaptive thresholding) and divided patients into 3 risk categories based upon those two inputs. Importantly, this algorithm does not use prespecified cut-points to derive risk groups; rather, the data are used to determine these groupings using an appropriate search algorithm and the results (Fig. 7) show, similar to earlier results (Figs. 4, 5 and 6), that the lowest 25% of the tumor-stroma interface F/B values identify a subgroup having the worst MFS experience. Furthermore, in the group having higher tumor-stroma interface F/B values (i.e., ln F/B ≥ 2.675) the S-ODX score subdivides this group into moderate (S-ODX ≥25.5) and low risk groups with respect to MFS. This regression tree approach does not deem tumor-stroma interface F/B calculated using the other two image analysis methods, nor any of the tumor bulk F/B measurements, as adding additional useful information in determining patient risk. The RSF algorithm does not produce a single tree; however, it produces measures of variable importance, and consistent with the regression tree approach, identifies F/B from the tumor-stroma interface calculated using adaptive thresholding and S-ODX as the two most influential predictors.

**Fig. 8:**
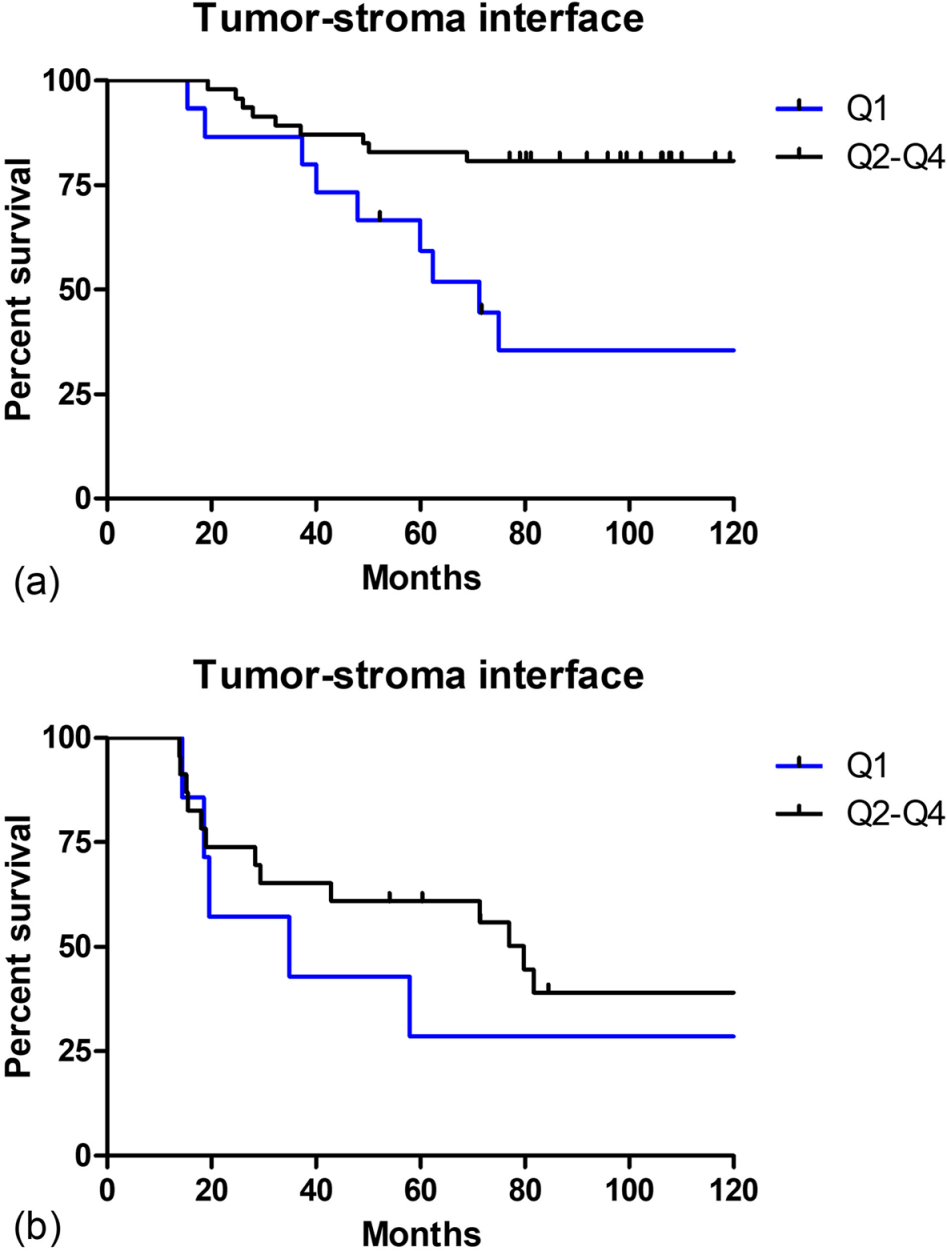
F/B is prognostic of MFS in the tumor-stroma interface of primary excisions with S-ODX **< 26.** Patients were divided by S-ODX score and then each group was split into four equal quartiles (Q1 = lowest F/B) based on F/B. The three higher quartiles (Q2-Q4) were pooled, and the percentage of each quartile surviving without metastasis then plotted versus time for these two groups (Q1 and Q2-Q4). **a**, S-ODX < 26 and **b**, S-ODX ≥26. Tick marks represent censoring events caused when a patient dies of a cause other than cancer or is lost to follow-up. Partial likelihood ratio test **a** *p* = 0.008, *n* = 62, **b** *p* = 0.4, *n* = 30

To determine if F/B can further stratify patient groups once their genomic score has been calculated, we next separated our patient cohort into two groups based upon their S-ODX score relative to the TAILORx cutoff (0–25 for low-intermediate and ≥ 26 for high-risk patients) [57]. We then generated Kaplan-Meier plots of ln F/B from tumor-stroma interface (calculated using adaptive thresholding) as described above (Fig. 8). Due to the low number of patients in each of the resulting graphs, we plotted the Q1 cohort versus the combined Q2-Q4 cohort to view the trends. F/B from tumor-stroma interface demonstrates prognostic ability in the S-ODX < 26 cohort, but not in the S-ODX ≥26 cohort (partial likelihood ratio test, *p* = 0.008 and *p* = 0.4, respectively).

## Discussion

Here we assessed the heterogeneity in F/B within IDC ER+ LNN patients, and the impact of that heterogeneity on possible use of F/B as a prognostic marker. We found that the heterogeneity in F/B within an individual tumor is not entirely random, as F/B measured in the cellular tumor bulk was statistically significantly different from F/B measured in the collagenous tumor-stroma interface. This agrees with our previously published study of core needle biopsy sections taken from IDC patients before NACT administration and subsequent tumor excision [48]. This suggests that the biological relevance of the collagen in the two tumor regions may be different, that the FIS of one region may have a different impact on metastasis than the other, and that the prognostic ability of F/B may vary between the two regions. Therefore, we assessed the relationship between SHG F/B and MFS in both these regions and, using user-defined thresholds to select collagen pixels for analysis, observed that F/B derived from the tumor-stroma interface has a stronger explanatory effect than does F/B from the tumor bulk.

Numerous automated image analysis and deep learning techniques have been developed to study breast cancer progression and improve diagnoses [61–63]. We implemented two image processing techniques with the goal of reducing user involvement in generating collagen fiber masks. Histogram-based thresholding (Otsu’s method) places pixels into foreground or background categories and finds the threshold between the groups that minimizes the sum of their variances. This reduces user input but does not account for heterogeneity in intensity within individual images. Adaptive thresholding compares each pixel’s value to the surrounding pixel average in a defined window. Because the window size is generally smaller than the image and is selected in an automated fashion for each individual image, this technique better preserves spatial variation and distinct contrasting features [55]. These methods also found the same relationships between F/B and metastatic outcome as F/B determined from a blinded user-defined threshold: for both methods, the combination of graphical analyses and Cox regression modeling found that F/B measured in the tumor-stroma interface had a stronger explanatory effect than F/B measured in the tumor bulk (Figs. 5, 6). This suggests that the difference in prognostic ability between the two regions is not due to the outcome-blinded observer setting thresholds in a different manner when faced with the two differently appearing types of regions, and confirms the impact of heterogeneity in the prognostic ability of F/B. Interestingly, in all image analysis methods F/B from the tumor-stroma interface appears to be identifying a cohort of patients (Quartile 1) with poor outcomes relative to the rest of the studied population.

We are interested in evaluating the possible use of F/B as a predictor of post-excision metastatic outcome, where it may inform the clinical decision-making process as to the nature of post-excision therapy. To further probe the effects of heterogeneity on the use of F/B for metastasis prediction, we evaluated the contribution of F/B from tumor-stroma interface and from tumor bulk on a survival prediction model. We developed models using both the Leblanc-Crowley regression tree and Random Survival Forest algorithms. These methods consider all input predictors and attempt to find the best prediction model using (possibly only some of) these inputs. One product currently used to assist in these treatment decisions is OncotypeDX®, a 21-gene score that is part of the current standard of care at our institution when planning post-excision treatment and is a known predictor of MFS [64]. Therefore, as inputs we included F/B from the tumor-stroma interface (3 image analysis methods), F/B from the tumor bulk (3 image analysis methods), as well as a 21-gene score inferred from Affymetrix data that is an economical surrogate for OncotypeDX® score [52], or 7 total inputs for each patient. The resultant regression tree (Fig. 7) finds that none of the three values of F/B from tumor bulk contributes to classifying patients based on risk. However, F/B from tumor-stroma interface (derived using adaptive thresholding) is first selected to split patients into groups based on recurrence risk, followed by S-ODX. The result is three risk groups: 1) patients with ln F/B < 2.65 (highest risk), 2) patients with ln F/B ≥ 2.675 and S-ODX ≥25.5, and 3) patients with ln F/B ≥ 2.675 and S-ODX < 25.5. This reveals that F/B from the tumor-stroma interface and S-ODX contribute to classifying patients’ metastatic risk, but that F/B from the tumor bulk does not, again demonstrating the effect of intratumoral heterogeneity on the possible use of F/B as a predictor of metastasis. Note that the selection of tumor-stroma interface F/B calculated using adaptive thresholding, and rejection of tumor-stroma interface F/B calculated using the other two methods, should not be interpreted as a strong endorsement of one image analysis method over the other, as the three are highly correlated (range 0.83–0.9) and hence are providing similar information. Interestingly, the regression tree split group 2 and 3 based upon an S-ODX value of 25.5, which is close to the TAILORx cutoff of 26.

Any tool to help predict metastasis and assist with treatment decisions is likely to be applied in combination with the now well-established genomic scores. To further understand how F/B can support genomic methods for guiding treatment decisions we divided our patient samples into two cohorts based upon the value of their S-ODX score relative to the TAILORx cutoff of 26 (separating low-intermediate and high-risk groups). Because our regression tree determined that F/B from tumor-stroma interface, but not bulk, is necessary to classify patients based on metastatic risk, we generated Kaplan-Meier plots of F/B from tumor-stroma interface (calculated using adaptive thresholding as it was selected by the RSF algorithm) (Fig. 8). F/B from tumor-stroma interface demonstrates prognostic ability in the S-ODX < 26 cohort, but not in the S-ODX ≥26 cohort (partial likelihood ratio test *p* = 0.008 and 0.4, respectively). The S-ODX < 26 cohort represents patients in the genomic low- and intermediate-risk groups, patients who will likely not be recommended for adjuvant chemotherapy. The F/B value from tumor-stroma interface appears to identify a subgroup of these patients with poor clinical outcome (Fig. 8). This suggests that after assessment of patients with a 21 gene risk score, F/B from tumor-stroma interface may be useful in further stratifying patients with low or intermediate recurrence scores, therefore providing a tool to better identify patients in need of adjuvant treatment, enrollment in clinical trials, or more intensive monitoring.

## Conclusions

Most breast cancer-related deaths are due to metastases. A tumor’s metastatic ability is affected by the microenvironment, including the extracellular matrix. The ratio of forward-to-backward-scattered (F/B) second-harmonic generation (SHG) photons can be used to study collagen fiber internal structure and has been shown to be an independent prognostic indicator of metastasis-free survival in invasive ductal carcinoma patients. These results demonstrate that, within a cohort of 95 untreated ER+ LNN IDC patients, intratumor heterogeneity has a significant impact on the possible use of F/B as a tool to predict metastatic outcome. They also suggest that F/B specifically from the tumor-stroma interface of primary tumor excisions may provide information, independent of cell-based morphology or genomic methods, to further stratify patients by metastatic risk and identify those in need of post-operative treatment. This assessment can be performed on the FFPE H&E slides already within the clinical workflow but, naturally, this implies that the slides used to determine F/B therefore contain tumor-stroma interface. Due to the low number of patient samples (*n* = 95), these results should be considered exploratory and they provide the impetus for additional research to confirm/replicate these findings.

## Supplementary Information

**Additional file 1: Table S1.** Primary tumor characteristics and associations with F/B. Primary tumor characteristics were measured and recorded after tumor resection, including progesterone (PgR) and human epidermal growth factor-2 (HER2) receptor expression, tumor stage, and tumor size in mm. Also shown is the association between these clinical variables and F/B from the tumor bulk and tumor-stroma interface produced using three analysis methods (individual thresholds, histogram-based thresholds, and adaptive thresholds) was assessed using Mann-Whitney tests (*p*-values listed).

**Additional file 2: Figure S1.** Full F/B heatmap and matching H&E for a representative primary tumor excision section. a) SHG F/B images (a series of adjacent ROIs extending along the *x*-axis) and b) matching H&E images were stitched end-to-end to form a composite ROI. These files are high definition versions of Fig. 1.

## Data Availability

The datasets used and/or analyzed during the current study are available from the corresponding author upon reasonable request. Genomic data for the patients used here are available from the GEO repository (in the GSE2034 and GSE5327 datasets) while the resultant S-ODX score is available from http://www.recurrenceonline.com/?q=GSE_download.

## Abbreviations

B: Backward-propagating SHG
ECM: Extracellular matrix
ER: Estrogen receptor
F: Forward-propagating SHG
FFPE: Formalin-fixed paraffin-embedded
FIS: Fiber internal structure
FITC: Fluorescein isothiocyanate
F/B: Forward-to-backward scattering ratio
H&E: Hematoxylin & eosin
IDC: Invasive ductal carcinoma
LNN: Lymph node negative
MFS: Metastasis-free survival time
RSF: Random Survival Forest
NACT: Neoadjuvant chemotherapy
ROI: Region of interest
SHG: Second-harmonic generation
S-ODX: Surrogate OncotypeDX® score
TAILORx: Trial Assigning Individualized Options for Treatment
