# Supplementary figures and images for: Intratumoral heterogeneity of second-harmonic generation scattering from tumor collagen and its effects on metastatic risk prediction
Source: BMC Cancer. 2020 Dec 10;20:1217. doi: 10.1186/s12885-020-07713-4 (PMC7731482; doi:10.1186/s12885-020-07713-4)

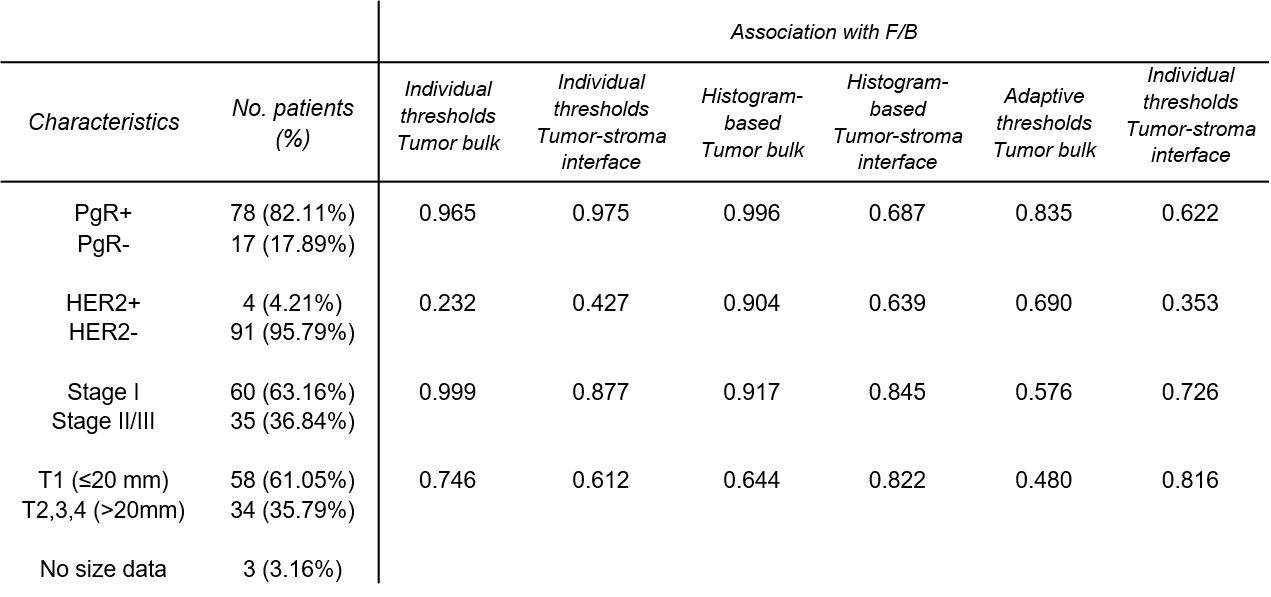

Supplement: Supplementary file 1 — Additional file 1: Table S1. Primary tumor characteristics and associations with F/B. Primary tumor characteristics were measured and recorded after tumor resection, including progesterone (PgR) and human epidermal growth factor-2 (HER2) receptor expression, tumor stage, and tumor size in mm. Also shown is the association between these clinical variables and F/B from the tumor bulk and tumor-stroma interface produced using three analysis methods (individual thresholds, histogram-based thresholds, and adaptive thresholds) was assessed using Mann-Whitney tests (p-values listed). [file 12885_2020_7713_MOESM1_ESM.png]

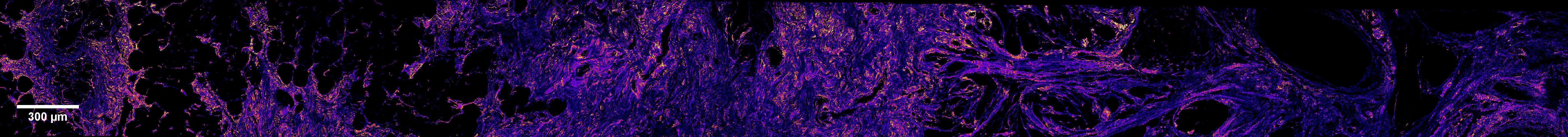

Supplement: Supplementary file 2 — Additional file 2: Figure S1. Full F/B heatmap and matching H&E for a representative primary tumor excision section. a) SHG F/B images (a series of adjacent ROIs extending along the x-axis) and b) matching H&E images were stitched end-to-end to form a composite ROI. These files are high definition versions of Fig. 1. [file 12885_2020_7713_MOESM2_ESM.zip › Supplementary_Fig_1aR2.png]

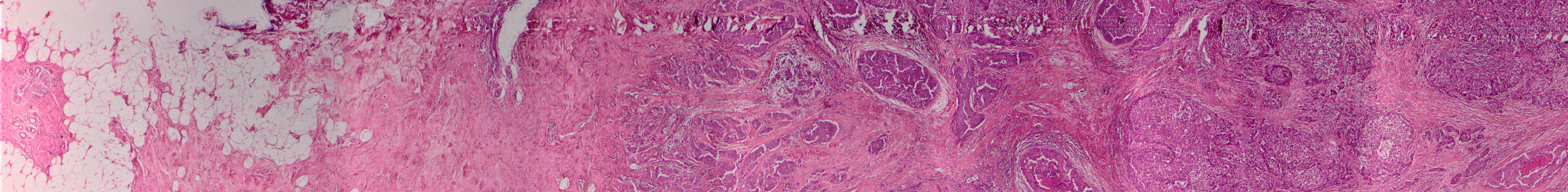

Supplement: Supplementary file 2 — Additional file 2: Figure S1. Full F/B heatmap and matching H&E for a representative primary tumor excision section. a) SHG F/B images (a series of adjacent ROIs extending along the x-axis) and b) matching H&E images were stitched end-to-end to form a composite ROI. These files are high definition versions of Fig. 1. [file 12885_2020_7713_MOESM2_ESM.zip › Supplementary_Fig_1bR2.png]
